# Efficacy and mechanisms of imagery rescripting and imaginal exposure for nightmares: study protocol for a randomized controlled trial

**DOI:** 10.1186/s13063-016-1570-3

**Published:** 2016-09-26

**Authors:** Anna E. Kunze, Jaap Lancee, Nexhmedin Morina, Merel Kindt, Arnoud Arntz

**Affiliations:** 1 University of Amsterdam, Nieuwe Achtergracht 129-B, 1018 WS Amsterdam, Netherlands; 2 LMU Munich, Leopoldstraße 13, 80802 Munich, Germany; 3 University of Münster, Fliednerstraße 21, 48149 Münster, Germany; 4 Amsterdam Brain and Cognition, Nieuwe Achtergracht 129, 1018 WS Amsterdam, Netherlands

**Keywords:** Nightmare disorder, Nightmares, Posttraumatic nightmares, Idiopathic nightmares, Imagery rescripting, Imagery rehearsal therapy, Imaginal exposure, RCT

## Abstract

**Background:**

Recurrent nightmares can effectively be treated with cognitive-behavioral techniques such as imagery rehearsal therapy, which involves imagery rescripting (IR) of nightmares, and imaginal exposure (IE) therapy. However, the underlying mechanisms of these treatments remain largely unknown. To investigate this, we identified a number of variables that might mediate the therapeutic effect of rescripting-based and/or exposure-based therapies. Also, to control for the possible confounding influence of (other) treatment components, we designed two stripped-down treatment protocols, which primarily consist of either (1) rescripting of, or (2) exposure to, the nightmare content. In a randomized controlled trial, we aim to investigate the therapeutic efficacy of these stripped-down IR and IE treatments, and explore their working mechanisms.

**Method:**

Three weekly sessions of either IR or IE will be compared to a waiting-list control group. Ninety participants suffering from nightmare disorder will be included and randomly allocated to one of the three groups. The primary clinical outcome measures are nightmare frequency and distress caused by nightmares. Secondary clinical outcome measures include sleep complaints, dysfunctional beliefs about nightmares, and posttraumatic stress symptom severity. Outcomes will be assessed weekly from week 1 (pre-assessment) to week 5 (post-assessment). Online follow-up assessments will take place at 3 and 6 months after post-assessment. In order to investigate temporal relationships between mediators and outcome, we will measure the proposed mediators of the treatment effect 1 day after each outcome assessment (but not after the follow-ups). Mediators include nightmare distress and valence, mastery of the nightmare content, predictability, controllability, and tolerability of emotions elicited by nightmares, as well as sleep quality.

**Discussion:**

The proposed trial allows us to investigate the efficacy of IR and IE as intervention techniques for the treatment of nightmares, and to explore mediators of their respective therapeutic effects. The results may advance our understanding of nightmare therapies by identifying possible mechanisms of psychological treatments for chronic nightmares. Moreover, the results of the proposed study might provide useful knowledge about the working mechanism of rescripting-based and exposure-based treatments in general.

**Trial registration:**

Netherlands Trial Register (NTR4951), registered on 14 December 2014.

**Electronic supplementary material:**

The online version of this article (doi:10.1186/s13063-016-1570-3) contains supplementary material, which is available to authorized users.

## Background

According to the fifth edition of the *Diagnostic and Statistical Manual of Mental Disorders* (DSM-5; [1]), nightmares are defined as extremely dysphoric dreams that typically involve threats to an individual’s survival and/or someone’s emotional or physical sense of safety, and they usually awaken the individual from sleep. Upon awakening, the individual quickly becomes alert and conscious of their surroundings. To qualify for the diagnosis “nightmare disorder,” nightmares need to cause substantial daytime suffering and distress. Typically, they are also accompanied by disrupted sleep and affective complaints. Furthermore, nightmares are often associated with various forms of psychopathology such as anxiety, depression, posttraumatic stress disorder (PTSD), suicidal ideation, substance abuse, and personality disorders [2–4].

The prevalence of nightmare disorder is high, with approximately 2–5 % of the general population suffering from one or more nightmares per week [5–7]. In a psychiatric sample, the prevalence rate was found to be much higher, with up to 30 % of patients suffering from nightmare disorder [8].

Several cognitive-behavioral therapy (CBT) techniques effectively ameliorate nightmare symptoms (for a review, see [3, 9]). To date, *imagery rehearsal therapy* (IRT) is the most empirically supported treatment for nightmares [9–11]. In IRT, patients are encouraged to change (rescript) the storyline of a nightmare into an alternative and less distressing story, which they then rehearse in their imagination (e.g., [12]). IRT can successfully decrease nightmare frequency and distress [10, 11], and it improves sleep quality [13, 14]. Next to IRT, *exposure* techniques are also effective in the treatment of nightmares. In imaginal exposure (IE) for nightmares, patients are confronted with and exposed to the content of their nightmares. Exposure-based treatment techniques have produced favorable changes in nightmare frequency and intensity in face-to-face [15, 16] and self-help formats [17–19].

Even though exposure-based and rescripting-based techniques both seem to be effective treatments for nightmares, there is an ongoing debate regarding the working mechanism of psychological treatments for chronic nightmares (see [11]). It has been argued that IRT and exposure may work via different pathways, since the techniques follow substantially different procedures. However, the working mechanisms of IRT and exposure for nightmares have not yet systematically been studied and, therefore, remain largely unknown. Thus, more research into the mechanisms of change in nightmare treatments is needed.

One way to gather knowledge about the working mechanisms of therapeutic techniques is to study variables that mediate treatment outcome [20, 21]. Such mediators are variables that may explain the relationship between an independent (e.g., treatment type) and a dependent variable (e.g., clinical outcome). Although mediators might not explicitly explain *how* behavior change occurs, they may uncover critical processes about *why* such change occurs [21]. Therefore, to further our understanding of the working of psychological nightmare treatments, the proposed study aims to investigate potential mediators of exposure-based and rescripting-based nightmare therapies. Possible mediators of the treatment effect are selected based on previous theoretical and empirical knowledge about exposure and rescripting treatments and/or their working mechanisms across psychological disorders.

### Mechanisms of change

Traditional theories of exposure therapy suggest that in order for the technique to be effective, exposure to a given stimulus needs to continue until the fear elicited by the stimulus eventually decreases. Within this emotional processing framework [22, 23], it is proposed that fear reduction (i.e., *reduction in Subjective Units of Distress*; SUDs) during and/or across exposure trials is a critical index of therapeutic change (but see [24] for a critical discussion of emotional processing theory). However, more contemporary models of exposure therapy do not emphasize fear reduction during or across exposure trials per se, but rather focus on other underlying processes. For example, inhibitory learning models of exposure therapy (e.g., [25, 26]) state that fear extinction (a laboratory analog for exposure therapy; [27]) does not erase a previously learned association between a stimulus (conditioned stimulus, CS) and an aversive event (unconditioned stimulus, US), but that it involves the formation of a new (inhibitory) memory (CS-noUS) while leaving the original fear memory (CS-US) intact [25, 28]. Inhibitory learning can be facilitated by certain processes that are assumed to be critical to successful exposure therapy [28], such as *fear toleration* [24]. While it is considered dysfunctional to down-regulate negative emotions through suppression or avoidance [24], experiencing the ability to tolerate such negative emotions during exposure (which is incompatible with the original fear memory) could stimulate new inhibitory learning [29]. In support of this proposition, it has been shown that lower tolerance of emotional distress is associated with higher PTSD symptom severity [30]. Moreover, avoidance of negative emotional states predicts severity of fear responding [31], whereas acceptance of negative emotions reduces distress [32].

Other variables that are often linked to extinction learning and exposure therapy include the controllability and/or predictability of aversive events [33]. The degree to which people perceive certain events to be within their control has long been suggested to be a fundamental mediator of psychopathology and treatment [34]. The significance of perceived controllability/predictability of aversive outcomes has, therefore, been acknowledged in numerous psychotherapy models (e.g., [35, 36]) as well as modern fear-learning theories (e.g., [37, 38]). Importantly, it has been proposed that not only uncontrollable aversive events, but also unexpected bursts of emotions may facilitate the development of anxiety disorders [35]. With regard to nightmares, the latter might be especially relevant, as patients regularly experience strong negative (and often fearful) emotions in response to their dreams. Thus, perceived controllability and/or predictability of harm (i.e., negative emotions as a result of nightmares) might be important variables in the treatment of nightmares. Though predictability/controllability of harmful events has foremost been associated with exposure therapy (e.g., [33]), it might also play an essential role in rescripting treatments. More specifically, in most rescripting-based therapeutic techniques, patients are encouraged to change a negative event according to their individual emotional needs. Hence, it can be assumed that patients gain control about the emotions they experience in response to a certain aversive event (e.g., nightmares). Therefore, we argue that *controllability and/or predictability* of emotions elicited by nightmares might be critical mediating variables in both IE and IR.

Even though empirical evidence regarding the underlying working mechanism of imagery rescripting (IR) is still limited, Arntz [39] recently proposed that IR might change the meaning of emotional events or memories through US devaluation, a process where fear memories or other aversive stimuli are degraded by *changing the negative valence* of such stimuli. There is preliminary evidence for the involvement of US devaluation in IR [40, 41], yet more research is needed to determine whether stimulus devaluation is a crucial mechanism for the efficacy of IR. Another variable that seems to play an important role in IR is mastery or self-efficacy. Research about IR for posttraumatic nightmares suggests that IR works by influencing negative beliefs about self-ability and the ability to control distressing images [42]. On a similar note, Krakow et al. [14] and Germain et al. [43] have argued that altering nightmares through IR techniques improves patients’ perceived mastery of nightmares (see also [11]). The hypothesis that increased mastery explains the effects of IR might be related to older explanations of IR, which emphasize the healing properties of expressing inhibited responses (see [39]). A key characteristic of nightmares is the inhibition of action tendencies in the dream, fuelled by the inability to control the muscles because of sleep paralysis, which leads to feelings of powerlessness and uncontrollability. By expressing these inhibited responses in the new script (e.g., attacking the perpetrator, successfully escaping from a dangerous situation, etc.) a feeling of mastery could be reestablished. Thus, *mastery of the nightmare content* might be an index of therapeutic change in IR.

Based on the above-mentioned theories and empirical findings, we identified the following variables as possible mediators of change for the treatment effect of IR and IE for recurrent nightmares: (1) nightmare valence, (2) predictability, (3) controllability, and (4) tolerability of emotions elicited by nightmares, (5) mastery of the nightmare content, and (6) reduction in SUDs. In addition, negative consequences of nightmares (i.e., distress caused by nightmares at night and during the day) are hypothesized to be a determining factor for nightmares to become recurrent and problematic [44]. Such nightmare distress (7) might, therefore, be an important mediator of the treatment effect for any nightmare therapy. Finally, we included sleep quality (8) in the set of proposed mediators for the treatment effect of IR and IE, as sleep plays a crucial role in the consolidation and reconsolidation of memories [45].

### Trial objectives

Investigating the role of these mediators in current nightmare therapies poses a methodological challenge, mainly because the treatments used to date consist of multiple treatment components. Even though IR [46, 47] and imaginal confrontation to the nightmare content [11] have been identified as the principal components of rescripting-based and exposure-based treatments, respectively, the most widely used formats of IRT and imaginal exposure for nightmares also incorporate nonspecific treatment modules. For example, IRT usually comprises rescripting and rehearsal exercises but also nightmare journals, safe place exercises as well as discussions of the nightmare content (i.e., exposure to the nightmare content; [12, 48, 49]). Similarly, exposure treatments often include imaginal safe place and/or relaxation exercises (e.g., [17]). Sometimes, exposure and rescripting are even directly combined ([42, 50, 51]). In sum, most nightmare treatments consist of a multitude of different treatment methods and components. Given that different treatment modules might target different types of symptoms [11], it remains challenging to extract the exclusive working of rescripting-based and exposure-based components within these treatments.

Against this background, we designed and developed treatment protocols, which consist mainly of either rescripting of, or exposure to, the nightmare content. Such stripped-down versions of exposure and rescripting for nightmares allow us to investigate the therapeutic efficacy of these treatments without any (possibly distorting) influence of nonspecific treatment components. While knowledge about the efficacy of pure rescripting and exposure for nightmares might be valuable to appraise the importance of different processes underlying psychological treatments for chronic nightmares, stripped-down treatment protocols have another significant advantage. Namely, using stripped-down versions of IE and IR in the treatment of nightmares may help to identify their mediating mechanisms. Thus, in order to (1) identify mediators of the treatment effect that (uniquely) contribute to the efficacy of IE versus IR, we aim to (2) establish the efficacy of each of these stripped-down treatments when compared to a waiting-list control group. In line with previous research, it is hypothesized that both treatments will effectively reduce nightmare symptoms [10, 11]. Potential differences between IR and IE will be explored. With regard to the mechanisms of change, we assume that both treatments target different key processes. This assumption is mainly based on current theories about the working mechanisms of IR and IE for nightmares. Specifically, we hypothesize that nightmare distress and tolerability of emotions elicited by nightmares might be critical mediating variables of the treatment effect of IE. In contrast, nightmare valence and mastery of the nightmare content might be particularly important mediators of the treatment effect in IR. However, given the limited empirical knowledge about the underlying mechanisms of nightmare treatments (especially IR), we cannot exclude the possibility that a number of the selected mediators might play a critical role in both treatments (e.g., predictability and controllability of emotions elicited by nightmares, and sleep quality), rather than being exclusive to either IR or IE. Therefore, we will further explore if the proposed variables play a unique role in either rescripting-based or exposure-based nightmare treatments, or whether they could be relevant mediators of therapeutic change in both therapies.

## Method

### Design

**Fig. 1:**
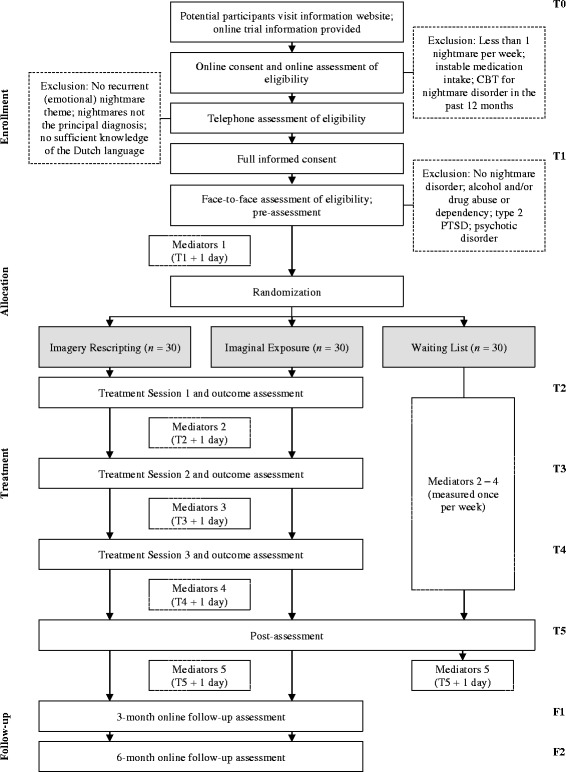
Proposed flow of participants. T1 = Pre-assessment, T2 = Week 1, T3 = Week 2, T4 = Week 3, T5 = Post-assessment, F1 = 3-month follow-up, F2 = 6-month follow-up

In this single-center randomized controlled trial (RCT) with three parallel groups, IR^1^ and IE therapy will be compared to a waiting-list control (WL) group. Participants are randomly allocated to one of three conditions: (1) IR, (2) IE, or (3) a WL condition, stratified by PTSD diagnosis. Participants in the WL condition receive one of the active treatments after a 5-week waiting period. The proposed mediators of change of the treatment effect will be explored in both therapies. For an overview of the proposed flow of participants, see Fig. 1. The present study protocol was written in accordance with the SPIRIT 2013 guidelines ([52, 53]; see Additional file 1 for an overview of the checklist items).

### Sample size

The proposed study was powered to detect differences between the treatments (i.e., IR and IE) compared to the WL condition. A power calculation (two-sided, power = 80 %, alpha = 0.05; G*Power 3.1) with a medium to large effect size for individual nightmare therapy (*d* = .74; [10]) showed that 30 participants in each condition would suffice to detect a statistically significant difference between each of the two treatment conditions and the WL condition. Thus, a total of 90 participants will be included in the proposed study and potential dropouts will be replaced.

### Eligibility criteria

Participants above the age of 18 will be included if they meet the following criteria: DSM-5 diagnosis of idiopathic and/or posttraumatic nightmare disorder [1]; one or more nightmare(s) per week; the nightmares have a recurrent (emotional) theme; and sufficient knowledge of the Dutch language. Exclusion criteria are: a current diagnosis of alcohol and/or drug abuse or dependency; PTSD resulting from protracted and recurring trauma (type 2 trauma); a current diagnosis of psychotic disorder; CBT-based psychotherapy for nightmare symptoms in the preceding 12 months; and unstable medication intake. Comorbidity as such will not be a reason for exclusion, but nightmare disorder must be the principal diagnosis. If applicable, participants will be asked to keep their medication intake stable during and 4–8 weeks before treatment (depending on the type of medication).

### Procedure

Participants will be recruited by means of advertisements in online social networks (i.e., Facebook, Twitter), a public website, and local newspaper announcements. Potential participants visit the information website where they will be provided with information about the trial, procedures, randomization process, confidentiality, and contact details. Interested participants fill out an online consent form and preliminary online screener, which assesses basic inclusion and exclusion criteria (e.g., availability of the participant, nightmare frequency and distress, as well as alcohol and drug intake). Based on this screener, eligible participants will be telephoned for a short interview, which aims at assessing nightmare symptoms, participant availability, possible medication intake, and differential diagnoses (e.g., pavor nocturnus). If participants appear to qualify for participation, they will be invited for a face-to-face intake interview. During the interview, a baseline (pre-)assessment of all outcome measures will take place. Then, written informed consent will be obtained from the participants and a member of the research team assesses each participant’s eligibility against all inclusion and exclusion criteria. Those who are not eligible for participation will receive an email outlining the reasons for exclusion. Participants who are eligible for participation will be randomly assigned to one of three conditions (i.e., IR, IE, or a WL control condition), and they will be notified of the randomization outcome via email or by telephone. To minimize the effects of knowledge about the treatment on all outcome measures and mediators, participants will only be informed that they are being allocated to “one of the treatment conditions” or “the waiting-list condition”, while the name (and nature) of the treatment is not communicated to them.

One week after baseline assessment, participants will receive the first of three treatment sessions (separated by at least 7 days). In the beginning of each treatment session, participants will be asked to fill out the primary treatment outcome measures (for an overview of the assessments, see Table 1). One week after the last treatment session or after a 4-week waiting period, post-assessment will take place. In order to ensure objective assessment of the treatment effect, an independent assessor, who is blind to the participants’ condition, will conduct the assessment. Proposed mediators of the treatment effect will be assessed by means of online questionnaires, which are sent via automated emails 1 day after pre- and post-assessment and after each treatment session. For participants in the WL condition, the online mediator questionnaires are sent 1 day after pre- and post-assessment, and once weekly during the waiting period (separated by 7 days). All face-to-face assessments and therapy sessions will take place at the outpatient psychotherapeutic clinic (PsyPoli) of the Department of Clinical Psychology at the UvA.

**Table 1:** Summary of measures

Measure	Description	T1	T2	T3	T4	T5	F1	F2
*Primary outcome measures*								
NFQ	Number of nightmares	+	+	+	+	+	+	+
NDIQ	Nightmare distress and impact	+	+	+	+	+	+	+
*Secondary outcome measures*								
NFQ	Nights with nightmares	+	+	+	+	+	+	+
ZIL	PTSD symptoms	+				+	+	+
ISI	Sleep complaints	+				+	+	+
NBQ	Nightmare beliefs	+				+	+	+
*Diagnostic measures*								
DSM-5	Nightmare disorder	+				+		
SCID-I	DSM-IV-TR Axis-I disorders	+				+		
*Mediators of change*		+	+	+	+	+		
*Imagery Exercise*		+	+	+	+	+		

*Note.* T1 = Pre assessment, T2 = Week 1, T3 = Week 2, T4 = Week 3, T5 = Post assessment, F1 = 3-month follow-up, F2 = 6-month follow-up. Mediators of change are assessed at T + 1 day. DSM-5 = *Diagnostic and Statistical Manual, 5th edition*; ISI = Insomnia Sensitivity Index; NBQ = Nightmare Beliefs Questionnaire; NDIQ = Nightmare Distress and Impact Questionnaire; NFQ = Nightmare Frequency Questionnaire; SCID-I = Structured Clinical Interview for DSM-IV-TR Axis-I disorders; ZIL = Zelf-Inventarisatie Lijst (Self-inventory List)

For all conditions, the full procedure spans 5 weeks in total. Participants in the waiting-list condition will receive one of the two treatments (by randomization) after the waiting period, but the treatment data of these participants will not be included into the main analyses. Three and 6 months after treatment, participants will be contacted by means of an automated email to complete the online follow-up questionnaires. Participants who do not complete the online questionnaires within 1 week will be contacted via personalized emails and/or telephone calls. When participants are unable to complete follow-up assessments online, a hardcopy of the assessment, including a return envelope, will be mailed to them. If participants decide to discontinue study participation, efforts will be made to retain them in the trial, while respecting their right to withdraw from participation at any time without any further consequences. Participants will not receive any monetary compensation for their involvement, but treatment will be delivered free of charge.

### Sequence generation and randomization

Treatment allocation will progress in accordance with an electronically generated allocation sequence (https://www.sealedenvelope.com/simple-randomiser/v1/lists). The size of the allocation blocks (i.e., 3, 6, and 9) is randomized, and eligible participants will be stratified according to a present PTSD diagnosis to ensure that participants with PTSD are distributed equally across conditions. The allocation sequence will be stored with two independent staff members who perform the randomization, and is concealed from all researchers, therapists, and participants.

### Measures

#### *Demographics*

Data on demographic variables such as age, gender, and educational level will be collected for all participants.

#### Diagnostic measures

Diagnosis of nightmare disorder will be based on criteria for nightmare disorder in the DSM-5 [1]. To determine the presence of current comorbid Axis-I disorders, the Dutch Structured Clinical Interview for DSM-IV-TR Axis-I disorders (SCID-I; [54]) will be administered at pre- and post-assessment. The reliability of the Dutch SCID-I is good, with a mean inter-rater agreement of .71 (Cohen’s Kappa; [55]).

#### Treatment outcome measures

Treatment outcome measures will be assessed at baseline, post-assessment, and 3- and 6-month follow-up. For an overview of all assessments, see Table 1.

#### Primary outcome measures

The first primary outcome measure addresses *nightmare frequency.* There are two ways to measure nightmare frequency with the widely used Nightmare Frequency Questionnaire (NFQ; [56]). This instrument assesses (1) the number of nights with nightmares in the last week, and (2) the total number of nightmares in the last week. As most previous nightmare research is focused on the latter, rather than the former conceptualization of nightmare frequency (e.g., [11, 13]), the total number of nightmares in the last week constitutes the first primary outcome measure, while the number of nights with nightmares in the last week is considered a secondary outcome measure. Both variables are assessed by means of a one-question, self-report survey (see NFQ; [56]).

The second primary outcome measure addresses *distress caused by nightmares* and the impact of nightmares on everyday life as well as on sleep behavior, which is assessed with a 12-item measurement instrument constructed for this study. For this purpose, several items of the Nightmare Distress Questionnaire (NDQ; [57]) and the Nightmare Effect Survey (NES; [58]) were modified and served as a basis for the new survey. This newly developed Nightmare Distress and Impact Questionnaire (NDIQ) consists of two subscales. The first subscale addresses the impact caused by nightmares during the day (e.g., “Because of my nightmares, I cannot function properly during the day”), while the second subscale is focused on the distress caused by nightmares at night (e.g., “My nightmares disturb my sleep pattern”). Given that nightmares do not only cause sleep complaints, but also severe daytime suffering, the authors aimed at constructing a questionnaire that could clearly distinguish these two sources of nightmare distress. Items of the NDIQ are scored on a four-point scale: 0 (Not), 1 (A little bit), 2 (Somewhat), and 3 (Completely). Validation of this questionnaire is still ongoing. The sumscore of both subscales constitutes the second primary outcome measure, while possible effects of IR and IE on the two subscales will be explored separately.

#### Secondary outcome measures

The Zelf Inventarisatie Lijst (ZIL; [59]) will be used to measure *symptoms of posttraumatic stress disorder*. The ZIL is a 22-item Dutch self-report inventory covering the severity of PTSD symptoms in the last 4 weeks. In contrast to other measures of PTSD symptoms, the ZIL allows for the assessment of posttraumatic symptoms irrespective of the occurrence of a traumatic event. Given that the proposed study aims to include participants with posttraumatic as well as idiopathic nightmares, it is assumed that (at least) some participants will not have experienced a traumatic event. The ZIL, therefore, seems to be a suitable measure of PTSD symptom severity in the present sample. The reliability of the scale is good, with Cronbach’s *α* varying from 0.90 to 0.94 [60], and a test-retest reliability of 0.92 [61].

*Sleep complaints* will be assessed with the Dutch version of the Insomnia Severity Index (ISI; [62]). The English questionnaire is a valid and reliable measure to detect changes in insomnia severity (internal consistency = 0.78; [63]). Validation studies on the Dutch version of this questionnaire are not yet published.

*Dysfunctional beliefs* about nightmares will be measured with a newly developed Nightmare Beliefs Questionnaire (NBQ). The NBQ was constructed based on nightmare interviews with four pilot participants of the proposed study. Given that dysfunctional beliefs have been shown to play an important role in sleep disorders (e.g., [64]) as well as other psychological disorders (e.g., [36, 65, 66]), it seems worthwhile to construct a measure that can assesses such dysfunctional beliefs about nightmares. The NBQ is a 15-item self-report questionnaire that measures the degree to which patients hold certain beliefs about nightmares (e.g., “People who suffer from nightmares are weak” or “I cannot control my nightmares”). Items of the NBQ are scored on a four-point scale: 0 (Not), 1 (A little bit), 2 (Somewhat), and 3 (Completely). Outcome values consist of the sum of all items, which can range from 0 to 45. The principal investigators (AK and JL) created the NBQ for the purpose of the proposed trial. Validation of this questionnaire is still ongoing.

#### Other measures

To evaluate and monitor changes in the type of emotions involved in nightmares over the course of treatment, participants will be presented with a selection of emotions commonly associated with nightmares (i.e., anxiety, anger, sadness, shame, disgust, guilt, and helplessness). They are asked to select the emotions they experienced during their nightmare(s) and encouraged to write down any other emotions they might have experienced.

If participants experienced one or more nightmares during the previous week, they will be asked to fill out four questions about the impact of the nightmare(s). On four Visual Analog Scales (VAS) ranging from 0 (“not at all”) to 100 (“very much”), participants are asked to indicate the intensity of emotions during the nightmare(s), the vividness of the nightmare(s), how often they awoke from nightmare(s), and their sleep quality during the past week (ranging from “very bad” to “very good”). In addition, all participants are asked to indicate their average hours of sleep during the past week. Note that all measures reported here will be assessed weekly.

#### Mediators of change

In order to investigate temporal relationships between the mediators and outcome measures in a methodologically well-considered manner [20, 67], the weekly appointments and assessments will be separated by a minimum of 7 and a maximum of 14 days. Proposed mediators of change for the treatment effect of IR and IE are assessed 1 day after each treatment session (and after pre- and post-assessment). Participants are asked to fill out an online questionnaire about their nightmares in general. This questionnaire consists of seven VASs ranging from 0 (“not at all” or “very bad”) to 100 (“very much” or “very good”), which measure nightmare valence, predictability, controllability, and tolerability of emotions elicited by nightmares, mastery of the nightmare content, sleep quality, and nightmare distress. Between-session reductions in SUDs will be assessed by means of the imagery exercise described below (for an overview of the proposed mediators and their corresponding items, see Table 2).

**Table 2:** Overview of proposed mediators

Mediator	Item
Predictability of emotions	“I think that I can predict the emotions elicited by my nightmares.”
Controllability of emotions	“I think that I can control the emotions elicited by my nightmares.”
Tolerability of emotions	“I think that I can tolerate the emotions elicited by my nightmares.”
Mastery of nightmare content	“I think that I am in control of the content of my nightmares.”
Nightmare valence	“When I think about my nightmares, I get emotional.”
Nightmare distress	“Nightmares have a negative influence on my daily functioning.”
Sleep quality	“How would you evaluate the quality of last night’s sleep?”
Reduction in SUDs	“How distressed do you feel right now?”

SUDs = Subjective Units of Distress

#### Imagery exercise

During baseline assessment, all participants will be asked to identify a core nightmare, which will later be addressed in treatment. Participants are instructed to choose a nightmare that is highly emotional and part of a recurring (emotional) nightmare theme (e.g., being killed, being followed, losing someone, etc.). The core nightmare is identified for several reasons: First, given that the present treatment consists of three 60-min treatment sessions only, it seems critical to identify the most distressing nightmare(s) as early as possible in the treatment process. Second, identifying and treating one particular nightmare allows for the investigation of nightmare-specific treatment effects. For this purpose, the core nightmare will be used in a weekly imagery exercise at the beginning of each treatment session. During the exercise, participants are asked to briefly imagine their core nightmare as vividly as possible until the emotions are sufficiently reactivated (approximately 1 − 3 min). Nightmare frequency, vividness, and SUDs, as well as tolerability of negative emotions elicited by the core nightmare, and the strongest emotion experienced at the moment of nightmare reactivation are being measured after nightmare reactivation. The nightmare used in this exercise is subsequently addressed in treatment. Note that while the exercise could be mistaken for exposure, the main goal of the imagery exercise is to *reactivate* the emotions sufficiently to address them in treatment, instead of prolonged exposure to these emotions (see also section “Imagery rescripting”).

### Interventions

Both interventions (IR and IE) are written out in a detailed treatment protocol that addresses the theoretical model, treatment frame, and the use of treatment techniques. The treatments consist of three individual 60-min sessions. Within these sessions, approximately 40 min will be spent on either exposure to or rescripting of the nightmare content. The remainder of the time will be used for filling out questionnaires (± 10 min), introducing and preparing the treatment exercises (± 5 min), and a short debriefing at the conclusion of each session (± 5 min). Cognitive-behavioral therapists with a completed Master’s degree-level education in clinical psychology will deliver both interventions. The therapists were trained by the authors of the proposed study (AK, JL, NM, and AA) during two 4-h training sessions. The training involved assessment and diagnosis of nightmare disorder, and a thorough explanation of the present treatment protocol, including sample treatments and exercises. After the waiting period in the WL condition, treatment will be delivered by trained but inexperienced therapists (baccalaureate degree-level). The authors (AK and JL) will supervise all therapists weekly. Treatment sessions will be audio-recorded and independent judges blind for treatment condition will rate a random selection of audiotapes on treatment adherence.

In order to monitor treatment progress, therapists will score participants’ nightmare vividness and SUDs, as well as tolerability of negative emotions elicited by the current nightmare image, and the strongest emotion experienced at the conclusion of each IE or IR exercise. For this purpose, therapists ask participants to indicate the strength/severity of the above-named variables on a scale from 0 (“not at all”) to 10 (“very much”) after each exercise. While the number and duration of IE and IR exercises within treatment sessions can differ across individuals and sessions (approximately two to four exercises during each treatment session), the total number and duration of individual treatment sessions will be the same between all participants (i.e., three 60-min sessions; approximately 40 min spent on IE/IR). Both interventions exclude homework assignments. Also, in order to minimize possible demand effects, the explanation of the treatment rationale is kept to a minimum (see Additional file 2, for standardized treatment rationales).

#### Imagery rescripting

The IR protocol used in the proposed study is inspired by the traditional IRT treatment protocols (e.g., [12]) as well as a protocol for IR therapy for early childhood trauma [68]. In contrast to more traditional IRT techniques, the present protocol focuses exclusively on IR exercises. More specifically, treatment components such as psychoeducation about sleep, nightmares, and mental imagery, as well as keeping nightmare diaries and discussing nightmare content were discarded. Instead, participants are introduced to the rescripting technique immediately at the beginning of the first treatment session. After reactivation of the core nightmare, participants are instructed to change the nightmare in any way they wish, as long as it leads to a satisfying story. In contrast to traditional IRT and in line with trauma-focused rescripting protocols (e.g., [68]), participants are asked to actively change the nightmare *in their imagination*. Thus, instead of first thinking about how to change the nightmare and subsequently rehearsing it, nightmares are changed directly after reactivation, while the accompanying emotions are still accessible. Activation of emotional memories seems to be necessary for the adequate integration of corrective information to occur [22]. Thus, in order for rescripting to be maximally successful, it has been proposed that the negative emotions accompanying an aversive event (e.g., nightmare) should be sufficiently reactivated before rescripting [39, 69]. It is important to discriminate between a short reactivation of emotions (approximately 1–3 min) and prolonged exposure, where patients are exposed to an aversive event repeatedly and for a longer period of time (usually 45–60 min). Thus, reactivation of an aversive event within rescripting treatments is *not* considered to be exposure, but a requisite component of the rescripting technique. Within the present protocol, it is not essential to rehearse a new nightmare script as often as possible, but rather to fine-tune the new script in such a way that the negative emotions accompanying the nightmare are maximally reduced until the participant is completely satisfied with the new script and eventually feels at ease. This can be accomplished within one single rescripting exercise, but it might also take multiple exercises (possibly across multiple sessions) in order for the participant to rescript the original nightmare in such a way that they feel entirely comfortable with the new nightmare script. Note that other nightmares than the core nightmare may only be addressed in treatment if rescripting of the core nightmare is successfully achieved early in the treatment process.

#### Imaginal exposure

The IE protocol used in the proposed study is based on a standard IE intervention [70]. Contrary to other exposure-based nightmare treatments, which are based on self-help formats and might include other treatment components such as nightmare diaries and relaxation exercises (e.g., [17, 19]), the current IE treatment consist of imaginal exposure to the nightmare content only. More specifically, after shortly reactivating the core nightmare, participants are asked to vividly imagine the entire nightmare scenario in their imagination and are encouraged to focus on and experience all accompanying emotions. If necessary, possible (cognitive) avoidance tendencies are shortly discussed to subsequently eliminate them. Similar to IR, participants receive approximately 40 min IE in total during each treatment session. However, individual IE exercises can differ in length depending on the nightmare scenario, the intensity of emotions elicited by the nightmare, and/or on the level that participants are willing to fully commit to the exercise. As for IR, exposure to nightmares other than the core nightmare is only allowed if exposure to the core nightmare has caused a substantial decrease of nightmare distress early in the treatment process, and if the negative emotions accompanied by the core nightmare are completely tolerable.

### Data management and storage

All study-related data and other study material will be stored securely at the study site (UvA PsyPoli). Participant information and study data will be kept in locked cabinets in areas with limited public access. After obtaining online informed consent, participants will be allocated a unique study identifier. A password-protected file that links participants to their identifiers is stored on a secure server hosted by the UvA. Any study material concerning participant information will not be released outside of the study without written permission of the participant.

Data collected on paper during the trial will manually be entered into a database. Self-report data collected online (using an authorized UvA Qualtrics account) will be downloaded and added to the database. Data integrity will be enforced through several ways, including valid values, range checks and consistency checks. The password-protected master database will be held on a secure server hosted by the UvA, where only authorized trial personnel have access to it. All obtained data and administrative forms (e.g., informed consent) will be stored in accordance with the data storage protocol of the UvA Department of Psychology (version January 2015).

### Statistical methods

#### Outcome

Treatment effects will be analyzed on the intention-to-treat principle with mixed regression. Fixed factors in the model will include: treatment condition, time, and their interaction, as well as any relevant covariate that might reduce error. The covariance structure of the repeated part will be determined by empirically assessing the best fitting structure. If possible, random parts on the participant level will be added. For the nightmare frequency outcomes, mixed Poisson or negative binomial regression will be used. For other outcomes standard mixed regression will be used, assuming a normal distribution of residuals. If residuals show a non-normal distribution, appropriate generalized mixed models will be used (e.g., gamma regression in case of skewed distributions).

#### Mediation

Mediators of the treatment effects for IR and IE will be analyzed according to the following steps: (1) mediator variables, which show a change during the course of treatment, will be included in the statistical model. For this purpose, the means across and within subjects over time will be inspected for all eight proposed mediators. In order to reduce the number of relevant mediator variables, we will inspect the association between the proposed mediators over time. A composite score will be calculated for those mediator variables that are highly associated with each other if their theoretical conceptualization allows for it. (2) In a series of separate mixed regression analyses, statistically relevant mediator variables will be selected from the remaining mediator variables. To that end, the effect of each mediator on treatment outcome will be explored regardless of treatment condition. Each mediator score (measured the day after pre-assessment and after all treatment sessions) will be used to predict treatment outcome at the following assessment (i.e., 6 days/nights later). (3) Only those mediator variables that have an effect on treatment outcome will subsequently be added to a final mixed regression model, taking treatment condition into account. Fixed factors in the model will, therefore, include: treatment condition (IE and IR), relevant mediators, time, and their interaction, as well as any relevant covariate that might reduce error. The covariance structure of the repeated part will be determined by empirically assessing the best fitting structure. If possible, random parts on the participant level will be added. Note that if a mediator represents a working mechanism, the strength of the association between mediator and effect (i.e., outcome) should be similar in all conditions [71]. Therefore, to investigate whether there is a difference between the working mechanisms of IR and IE, we will test whether the mean change for each mediator differs between treatment conditions (i.e., the larger the change, the more the treatment affects the mediator, indicating that the working mechanism is strongly triggered by the treatment). Also, we will inspect the percentage of variance of the clinical effect explained by each mediator, and whether treatments differ in the percentage of variance explained by a specific mediator. (4) In a last step, all previously determined relevant mediator variables will subsequently be tested with separate mediation analyses for IE and IR (with WL as a reference group). Difference scores of mediator assessments will be used to predict post-assessment outcomes. Pre-treatment levels of the dependent and mediator variables will be added to the model as covariates (see also [64]). Both single and multiple mediation analyses will be performed. Mediation will be tested by evaluating the 95 % confidence interval of the indirect effect (i.e., the effect of the intervention (IE or IR) on treatment outcome through a specific mediator). For these analyses, we will use bootstrapping [72, 73], a nonparametrical procedure that produces an estimate of the sample distribution based on several resamples. For the proposed analyses, a minimum of *n* = 5000 bootstrap resamples will be generated. To the best of our knowledge, bootstrap mediation is currently not available for mixed regression. Unless such a technique is available by the time of the analyses, we will execute the bootstrap mediation test on the subsample with complete data, as well as on the complete sample with missing values imputed, to check for consistency of results. A multiple imputation procedure based on 20 datasets will be used to replace missing values.

## Discussion

With the proposed RCT, we aim to explore mechanisms of change in rescripting-based and exposure-based treatments for nightmare disorder. For this purpose, we introduce adapted IR and IE treatment protocols for nightmare disorder, each including one primarily active treatment component (i.e., either rescripting of, or exposure to, the nightmare content, respectively). Next to investigating the efficacy of these stripped-down treatments when compared to a waiting-list control condition, the research design of the proposed study enables us to explore proposed mediators of the treatment effect for IR and IE. Studying mediators can offer important knowledge about the underlying working mechanisms of psychological treatments, which might improve the effectiveness of everyday clinical practice.

The study has several methodological strengths. First, it follows the standard for evaluating the efficacy of psychological treatments (e.g., randomization of participants to three conditions, including a waiting-list control condition, allocation is concealed by means of randomization by independent staff members, post-assessment conducted by blinded researchers). Second, the current research design is well-suited to study mediators of change for the treatment effect of IR and/or IE for nightmare disorder, which might be a first step toward identifying possible mechanisms of change for these treatments [20]. More specifically, measuring several potential mediators simultaneously at multiple time points throughout the trial (i.e., preceding the intermediate treatment effect assessments) allows us to establish temporal as well as causal relationships between proposed mediators and therapeutic outcome measures [20, 21]. Another advantage of the proposed study is its use of stripped-down treatment formats. This method enables us to gather relevant information about the efficacy of specific treatment techniques (i.e., IR and IE) rather than nonspecific treatment components (e.g., nightmare journals, relaxation exercises, extensive psychoeducation about sleep and nightmares). Accordingly, the proposed trial does not aspire to design the most efficacious treatment package for nightmare disorder, but it rather aims to investigate the efficacy of the separate treatment components, and to explore differences and similarities in efficacy and working mechanisms between rescripting-based and exposure-based nightmare treatments. In order to draw conclusions about the active component of nightmare therapies, we find it important to study the two treatment techniques in isolation. Once we have examined the two techniques separately, future studies should concentrate on creating the optimal treatment package for nightmare disorder.

The proposed study also has a number of weaknesses. First, consistent mediator assessment might be compromised by the logistical complexity of the study. More specifically, while we aim at assessing treatment outcome variables and mediators every 7 days, appointments might sometimes be cancelled and/or rescheduled. In order to maintain the temporal relationship between mediators and outcome variable measurements as stable as possible, appointments may only be (re)scheduled to a *later* time point. Second, due to practical considerations, the proposed study has a relatively small sample size. Therefore, its statistical power might be too small to detect statistically significant differences between IE and IR with regard to treatment efficacy and/or mediators of change. However, exploring potential differences between IE and IR might inspire future research in this domain. Third, the waiting-list period of the proposed study is rather short. As a consequence, we cannot assess the long-term effects of the stripped-down treatments. However, if the results of the proposed trial support the efficacy of both interventions, future research might directly compare the two techniques as described in the present treatment protocol to a waiting-list condition with a longer waiting period. Fourth, two of the questionnaires used in the present study have not yet been validated (NDIQ and NBQ). It should be noted that the outcome variables used in nightmare treatment studies often lack precise definitions. With regard to nightmare research, it has become customary to assess nightmare frequency and nightmare distress. Such nightmare distress can be measured either in terms of distress directly associated with nightmares (e.g., NDQ [57]), or in terms of the effects nightmares have on a person’s life (e.g., NES [58]). While these questionnaires tap into different constructs of nightmare distress, they also show some considerable overlap. Therefore, we decided to create a nightmare distress outcome measure (i.e., NDIQ), which combines different aspects of nightmare distress in a single questionnaire. We acknowledge that a lack of validation might reduce confidence in the NDIQ as an outcome measure. However, the NDIQ is constructed from items resembling those of two validated questionnaires (NDQ and NES). Given that the items included in the NDIQ are very similar to those from these other questionnaires, we expect the NDIQ to be a valid and reliable measure. As to the NBQ, it should be mentioned that dysfunctional nightmare beliefs have not systematically been studied to date. The NBQ, a novel questionnaire altogether, aims to assess the occurrence of dysfunctional nightmare beliefs in nightmare sufferers. It was constructed to resemble other validated dysfunctional beliefs questionnaires (e.g., Dysfunctional Beliefs and Attitudes about Sleep [74]; Panic Belief Inventory [75]; Metacognitions Questionnaire [76]). To assess the psychometric properties of the NDIQ and NBQ, prior to data analyses, both questionnaires will be validated within a representative sample of nightmare sufferers and healthy controls. Fifth, the selected mediators are assumed to play a crucial role in IE, IR, or both. While we acknowledge that there are a multitude of (other) proposed theories and processes underlying IE and IR, we focus on those variables that have recently received much attention in the empirical literature and that seem especially relevant in the treatment of nightmares. Accordingly, it is possible that other relevant processes or variables were disregarded.

Taken together, the proposed project offers a unique opportunity to investigate the efficacy of two core treatment techniques for nightmares (IR and IE), and to explore and identify their mechanisms of change. More specifically, by including mediator and outcome variable assessments at multiple time points throughout the study, we intent to investigate temporal relationships between changes in mediators and their effects on outcome measures, which are needed to determine causal pathways of therapeutic change. Thus, with the proposed trial, we aim to provide new insights in the mechanisms of change of IR and IE for nightmares and thereby contribute to the improvement of such treatments. Moreover, given that rescripting as well as exposure techniques are also used in the treatment of other disorders, the results of the current study might provide useful knowledge about the working mechanism of these therapeutic techniques in general.

### Dissemination of results

The results of the proposed study are intended for publication in peer-reviewed journals independently of the outcome of the trial. The scientific output of the proposed study will include at least one paper about the efficacy of IE and IR, and one paper about the mechanisms of therapeutic change. Participants will be informed about the results of the study after publication by means of a Dutch summary of the results.

### Trial status

The first participant was enrolled in January 2015. Participant recruitment and enrollment is currently in the final stage and is expected to continue until May 2016. Data collection will proceed until the end of 2016.

## Additional files

Additional file 1: SPIRIT 2013 checklist. (PDF 121 kb) Additional file 2: Treatment rationale of imaginal exposure (IE) and imagery rescripting (IR) for nightmares. (PDF 67 kb)

## Abbreviations

APA: American Psychological Association
CBT: Cognitive-behavioral therapy
CS: Conditioned Stimulus
DSM-IV: *Diagnostic and Statistical Manual of Mental Disorders, 4th edition*
DSM-5: *Diagnostic and Statistical Manual of Mental Disorders, 5th edition*
IE: Imaginal exposure
IR: Imagery rescripting
IRT: Imagery rehearsal therapy
ISI: Insomnia Severity Index
NBQ: Nightmare Beliefs Questionnaire
NDQ: Nightmare Distress Questionnaire
NDIQ: Nightmare Distress and Impact Questionnaire
NES: Nightmare Effects Survey
NFQ: Nightmare Frequency Questionnaire
PTSD: Posttraumatic stress disorder
RCT: Randomized controlled trial
SCID-I: Structured Clinical Interview for DSM-IV-TR Axis-I disorders
SUD: Subjective Unit of Distress
US: Unconditioned stimulus
UvA: University of Amsterdam
VAS: Visual Analog Scale
WL: Waiting-list
ZIL: Zelf-Inventarisatie Lijst (Self-inventory List)

## Footnotes

1 For the remainder of this article, the stripped-down rescripting-based nightmare treatment will be called IR to conceptually distinguish it from the currently widely used imagery rehearsal therapy (IRT).
