# Supplementary material for: Efficacy and mechanisms of imagery rescripting and imaginal exposure for nightmares: study protocol for a randomized controlled trial
Source: Trials. 2016 Sep 26;17:469. doi: 10.1186/s13063-016-1570-3 (PMC5037644; doi:10.1186/s13063-016-1570-3)
Supplement: Additional file 2: — Treatment rationale of imaginal exposure (IE) and imagery rescripting (IR) for nightmares. (PDF 67 kb) [file 13063_2016_1570_MOESM2_ESM.pdf]

Efficacy and Mechanisms of Imagery Rescripting and Imaginal Exposure for Nightmares: Study Protocol for a Randomized Controlled Trial

Additional File 2

Anna E. Kunze[1,2], Jaap Lancee[1], Nexhmedin Morina[3], Merel Kindt[1,4], and Arnoud Arntz[1]

[1]University of Amsterdam, Nieuwe Achtergracht 129-B, 1018 WS Amsterdam, Netherlands

[2]LMU Munich, Leopoldstraße 13, 80802 Munich, Germany

[3]University of Münster, Fliednerstraße 21, 48149 Münster, Germay

[4]Amsterdam Brain and Cognition, Nieuwe Achtergracht 129, 1018 WS Amsterdam, Netherlands

Treatment Rationale of IE and IR for Nightmares

In the beginning of the first treatment session, therapists read the following treatment rationales to patients:

Imaginal Exposure. "As you know, your nightmares are associated with extremely negative emotions. During the treatment, we will try to untighten the connection between these emotions and the nightmares. We are going to achieve this by imagining the nightmare as detailed as possible. This means that we are going to deliberately evoke the nightmare and focus on the negative emotions it elicits. I realize that this might be very unpleasant, but by purposely not trying to avoid or repress the nightmare, you will get better and the nightmare will become less intense. We know from previous experience that your nightmares will not only get less intense, but that they will occur less frequently."

Imagery Rescripting. "As you know, your nightmares are associated with extremely negative emotions. During the treatment, we will try to untighten the connection between these emotions and the nightmares. We are going to achieve this by shortly reactivating the nightmare and subsequently changing it. I will provide an explanation for 'changing the nightmare' later. By reactivating the nightmare and then changing it, the connection between your nightmare and the associated negative emotions is loosened, and the nightmare will become less intense. We know from previous experience that your nightmares will not only get less intense, but that they will occur less frequently."

If patients are dissatisfied with the IR or IE rationale or have more questions about the treatment, therapists are instructed to say the following: "During the therapy, we will make use of imagery exercises. We know that visually imagining and processing negative events is more effective than just talking about them. For example, we know that imagining a certain event activates the same brain regions as actually experiencing the event or situation (even if

you know the situation is not real). That is why imagery exercises are especially well suited to treat nightmares."

[Translated from the Dutch by AK and JL]
